# Out-of-Hospital Cardiac Arrest in the Paediatric Patient: An Observational Study in the Context of National Regulations

**DOI:** 10.3390/jcm13113133

**Published:** 2024-05-27

**Authors:** Roberta Pireddu, Giuseppe Ristagno, Lorenzo Gianquintieri, Rodolfo Bonora, Andrea Pagliosa, Aida Andreassi, Giuseppe Maria Sechi, Carlo Signorelli, Giuseppe Stirparo

**Affiliations:** 1 School of Public Health, Faculty of Medicine, University Vita-Salute San Raffaele, 20132 Milano, Italy; 2 Department of Medical and Surgical Pathophysiology and Transplantation, University of Milano, 20133 Milano, Italy; 3 Fondazione IRCCS Ca’ Granda Ospedale Maggiore Policlinico, 20090 Milano, Italy; 4 Department of Electronics, Information and Bioengineering, Politecnico di Milano, 20133 Milano, Italy; 5 Agenzia Regionale Emergenza Urgenza Headquarters (AREU HQ), 20124 Milano, Italy

**Keywords:** epidemiology, public health, policy, OHCA

## Abstract

**Introduction:** Cardiac arrest results in a high death rate if cardiopulmonary resuscitation and early defibrillation are not performed. Mortality is strongly linked to regulations, in terms of prevention and emergency–urgency system organization. In Italy, training of lay rescuers and the presence of defibrillators were recently made mandatory in schools. Our analysis aims to analyze Out-of-Hospital Cardiac Arrest (OHCA) events in pediatric patients (under 18 years old), to understand the epidemiology of this phenomenon and provide helpful evidence for policy-making. **Methods:** A retrospective observational analysis was conducted on the emergency databases of Lombardy Region, considering all pediatric OHCAs managed between 1 January 2016, and 31 December 2019. The demographics of the patients and the logistics of the events were statistically analyzed. **Results:** The incidence in pediatric subjects is 4.5 (95% CI 3.6–5.6) per 100,000 of the population. School buildings and sports facilities have relatively few events (1.9% and 4.4%, respectively), while 39.4% of OHCAs are preventable, being due to violent accidents or trauma, mainly occurring on the streets (23.2%). **Conclusions:** Limiting violent events is necessary to reduce OHCA mortality in children. Raising awareness and giving practical training to citizens is a priority in general but specifically in schools.

## 1. Introduction

Cardiopulmonary Arrest is defined as the cessation of mechanical activity of the heart. When it occurs outside of a healthcare facility, it usually referred to as Out-of-Hospital Cardiac Arrest (OHCA), whether it is generated by cardiac or non-cardiac causes [1]. This condition is irreversible if bystanders or prehospital emergency responders do not intervene promptly through Basic Life Support (BLS) maneuvers and with the use of an Automated External Defibrillator (AED) [2], according to the guidelines and the resources provided by Public Access Defibrillation (PAD) programs.

BLS maneuvers depend on a patient’s age, and different guidelines were implemented for pediatric and adult patients [3]. However, studies have focused on adult OHCA, because the incidence in adults (about 50–150 yearly events per 100,000 inhabitants [4] in European countries) is much higher than in pediatric patients (about 8 cases per 100,000 inhabitants [4]). Studies on this population in the Italian national territory have yet to be completed. However, interest in the topic was raised by Law No. 116 of 4 August 2021, which indicates AED installation and BLS training as mandatory in schools and describes a phased implementation protocol [5]. This reform emphasizes the importance of training not only for health professionals [6] but also the lay population [7,8], with training sessions organized in schools and public places [9].

An epidemiological analysis is vital for evidence-based standard setting and a policy implementation that is in line with the real needs of the population [10,11], and a centralized and data-driven decisional process for the deployment of AED can substantially increase the performance of PAD programs [12]. For this reason, a focus on pediatric cardiac arrest is necessary for the Italian territory, now more than ever, considering the recent increase in interest in the topic [13]. In addition, it may be helpful to compare local trends with the results of studies conducted in other countries. In this way, it can be assessed whether the incidences are aligned and whether the Italian emergency–urgency (E-U) system succeeds in guaranteeing, for pediatric patients, the outcome standards in terms of the probability of Return of Spontaneous Circulation (ROSC) [14,15,16,17]. This aspect is particularly relevant when also taking into consideration the different variables that influence outcomes for patients [18,19,20].

In Lombardy, prehospital emergency management is entrusted to the AREU (Regional Agency for Emergencies and Urgencies), which coordinates all the emergency requests from the regional territory. If the control center technician identifies an emergency possibly involving a patient in cardiac arrest, according to AREU protocols and operational instructions, the central operator immediately dispatches an advanced medical team (MSA) with a red code (highest priority). Pre-arrival instructions (IPA) are also provided to the bystander who made the call, with the aim of enabling the delivery of immediate cardiopulmonary resuscitation (CPR) to the patient in OHCA [21,22]. 

The COVID-19 pandemic significantly affected the Lombardy E-U system [23,24,25], leading to changes in time-dependent disease networks [26,27], particularly impacting OHCA [28,29,30]. These changes hindered research reliability in the pre-pandemic phase, and studies of this kind aim to resume evidence-based decision systems, not focusing solely on COVID-19, which temporarily interrupted research on time-dependent diseases [31]. It is worth noticing that studying time-dependent diseases still relies on pre-pandemic data, such as disease registries and incidence rates. 

Lay training retains a central role in the proper management of OHCA emergencies [32,33,34], in parallel with a proper development of the E-U system, which is just as much critical [35,36]. Therefore, the aim of this study was to conduct a joint epidemiological analysis of OHCA in pediatric subjects, involving all the stakeholders concerned with pediatric cardiac arrest, considering as the study case the area of the Lombardy region, in northern Italy, in the time period before the COVID-19 pandemic. Starting from the analysis of the epidemiological context and inspecting the dynamics (the location of the event and type of intervention), the ultimate goal is to assess the phenomenon and the real needs of the population in the context of implementing Law No. 116 of 4 August 2021 [5].

## 2. Materials and Methods

This is a retrospective observational study. It was conducted in accordance with the principles of the Declaration of Helsinki and was approved by the Data Protection Officer (DPO) of AREU. As such, the data included in the analysis were anonymized in origin, therefore no informed consent could be obtained from individual patients; yet, no dis-aggregated statistic was provided that could possibly allow identification.

The data from all the missions managed by the Emergency Medical Services (EMS) in the Lombardy region and registered as Cardiocirculatory Arrest in the AREU database from 1 January 2016 to 31 December 2019 were analyzed. The collected data were relevant to the patient’s age, sex, time of the event, location (geographic coordinates and type of location), rescue vehicles involved (and their time of arrival on scene), and presumed cause of the arrest, along with Utstein model data about the event and the intervention.

The first output were basic statistics, oriented to depict the phenomenology of pediatric OHCA in the target territory during the analysis period. Patients less than or equal to 18 years of age were considered pediatric. Such definitions may vary in different contexts; however, the term ‘children’ as intended in the current study refers to this part of population, as opposed to ‘adult’. Categorical variables were presented with absolute values and percentages, and continuous variables were presented with mean and standard deviation. The significance of the categorical variables was calculated with a *p*-value < 0.05, and the CI was defined as 95%.

In particular, the data analysis focused on the following: (I) the distribution of the events across the territory, in terms of prevalence and incidence, (II) the age of the patients, distinguishing pediatric and adult populations and assessing age distribution in the first class, (III) the etiology of the events, not only distinguishing between cardiac and non-cardiac ones but also specifying the different causes leading to non-cardiac ones, (IV) the place of the events, as classified by EMS personnel intervening, and (V) the outcome and rescue treatment (also in relation to different places of occurrence).

A second output was instead the assessment of the significance of the different occurrence frequencies of certain conditions related to the event (witnessed or not, type of intervention performed, and outcome) in relation to the different locations or causes of the arrest. For this purpose, the univariate odds ratio (OR) was computed, separating the two populations on the basis of the location/cause (targeting one against all others), and setting the different conditions as the output target. In these cases, an OR > 1, with C.I. 95% values both >1, corresponds to a positive correlation (the targeted location/cause having an increased frequency of occurrence of the target condition), while an OR < 1, with C.I. 95% values both <1, corresponds to a direct negative correlation (the targeted location/cause having a decreased frequency of occurrence of the target condition). Regardless of the central OR value, when C.I. 95% values are, respectively, below and above 1, and when the *p*-value is >0.05, no conclusive evidence can be drawn about the impact of the targeted location/cause on the target condition.

## 3. Results

**Figure 1:**
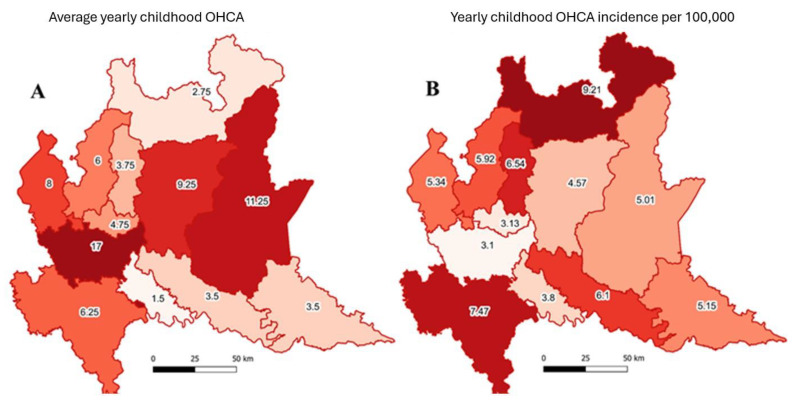
Average yearly number (from 2016 to 2019) of childhood (<18 y.o.) out-of-hospital cardiac arrests in different provinces of Lombardy region, Italy (**panel A**), and relevant incidences (**panel B**) on target population (number of events/100,000 residents < 18 y.o.).

Out of a total of 46 343 cardiac arrests recorded in the AREU database, 315 events involved people under the age of 18 years, accounting for 0.7% of the total. Of these, 109 (34.6%) were female, and 195 (61.9%) were male; in the remaining 11 (3.5%), sex was not included in the records. The distribution and incidence of these events are reported in Figure 1. The number of events is not uniformly proportional to the resident population, resulting in sparse incidence, with a three-fold ratio between the extremes (3.1 against 9.21), respectively, recorded in the most urbanized and most densely populated area (min value) and in the most natural and least densely populated one (max value).

**Figure 2:**
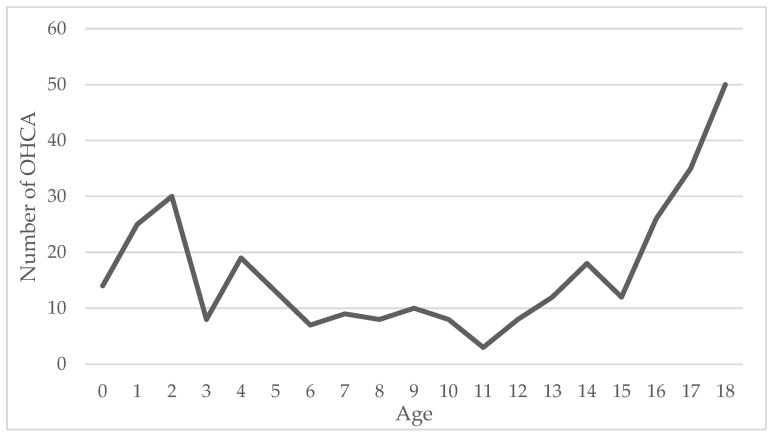
The number of pediatric OHCAs by age, which occurred in the Lombardy region, Italy, between 1 January 2016 and 31 December 2019, and rescued by the emergency–urgency system.

**Figure 3:**
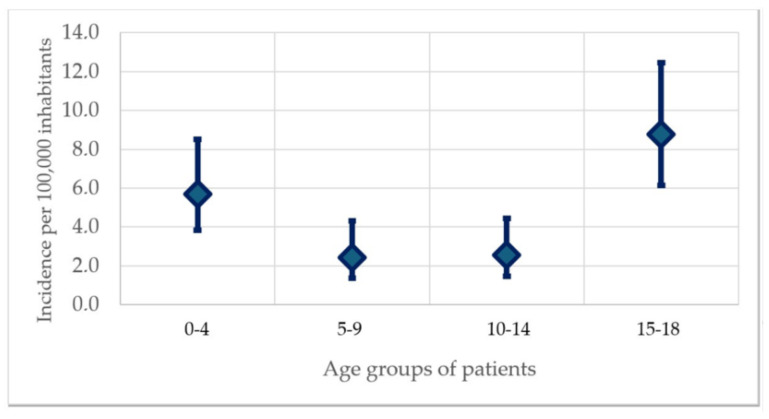
The incidence of pediatric OHCAs occurring in the Lombardy region, Italy, between 1 January 2016 and 31 December 2019 and rescued by the emergency–urgency system, in different age groups with 95% CI.

Regarding the age of the pediatric patients, the mean was 10.0 (SD: 6.0) years. The absolute number of OHCA in pediatric age has a bi-modal pattern, with peaks at ages 0–4 years and 15–18 years, as emerges from Figure 2. Within the context of our study, the incidence of OHCA in pediatric age had a value of 4.5 (95% CI 3.6–5.6) per 100,000. This incidence had higher results at the extremes of the age groups examined, confirming the bi-modal distribution: it was 5.7 (95% CI 3.8–8.5) in the 0–4 age group, while in the 15–18 age group, it was 8.8 (95% CI 6.5–12.5), as reported in Figure 3.

The reasons behind pediatric OHCAs were investigated (with the complete results reported in Table 1). In 174 cases (55.2%) only, the cause was an acute medical event. In 124 cases (39.4%), the identified cause can be considered preventable, being unrelated to a medical condition, such as violent events or accidents. In the remaining 17 cases (5.4%), the cause is unknown as not reported in the database.

With regards to the place where the events occurred, most of them (58.7%) were at home, while the second most common place was the street (23.2%). Schools recorded 6 events (1.9%), while 14 (4.4%) occurred in sport facilities. It is worth noticing that the total number of events which occurred in schools in the study period is 22, meaning that, in such locations, less than one event out of three affects minors, while two out of three regard adults. The complete results are reported in Table 2.

The data relevant to the rescue response context, as for the Utstein model, are reported in Table 3. CPR was performed by lay personnel in 113 (35.9%) cases out of the total pediatric OHCAs, 74 of which occurred at home and 13 at sports facilities. Resuscitation at home was more likely than in other locations (OR: 1.8; 95% CI 1.2–3.0; *p* = 0.01), but the highest probability was achieved in sports facilities (OR: 8.6; 95% CI 2.4–39.9; *p* = 0.001). Within schools, the data are too scarce to make assumptions about the probability of receiving CPR; in fact, only six subjects are present in the database, three of whom received CPR (OR: 2.0; 95% CI 0.4–10.5; *p* = 0.37). PAD (Public Access Defibrillation) was performed in 10 cases only (3.2%), in relation to events occurring at home (3), at a sports facility (3), on the street (3), and at school (1). According to this analysis, the probability of receiving defibrillation in a sports facility is higher than in other places (OR: 12.0; 95% CI 2.7–52.7; *p* = 0.001), while in school, the data are once again too scarce, with only 1 in 6 subjects receiving a PAD (OR: 6.6; 95% CI 0.70–63.07; *p* = 0.10). 

In relation to the first presentation rhythm, 215 (68.2%) patients had a non-shockable rhythm, 28 (8.9%) had a shockable rhythm, and in 72 (22.9%) cases there was no rhythm of presentation in the rescue mission summary.

An MSA was dispatched in 308 events, accounting for 97.7% of the total pediatric OHCA missions; in these missions, PALS maneuvers were performed in 212 cases (67.3%). The ECMO code (eligibility for Extracorporeal Membrane Oxygenation procedure) was indicated for 14 patients only.

## 4. Discussion

The percentage of pediatric OHCAs managed by the EMS in the Lombardy Region over the four years of the analysis was 0.7% of the total. A total of 20% of the patients reached ROSC before arrival at the hospital, which is in line with the analysis published by Irvine R. et al. [16]. Figure 1 illustrates a notable trend: pediatric OHCA incidence was higher in less urbanized and non-metropolitan areas.

Table 2 shows the location of the patient encounters. It is worth noticing that the share of patients rescued at home (58.7%), despite being the majority, is consistently lower than for adult subjects (85%). As presented in our previous studies, this could be related to the fact that OHCA often affects elderly subjects, who tend to spend most of their time at home, more than younger patients. More relevantly, we must point out that many OHCAs occur on the street, which could be either related to traffic accidents or, instead, may be unrelated to a violent dynamic and simply occurring during everyday outdoor activities. To this end, we have highlighted the main reason, as reported by EMS personnel intervening on the scene for OHCA in Table 1, and a very relevant finding, that deserves the attention of all childcare stakeholders, emerges by cross-analyzing two parts of information. Among all pediatric OHCAs, 8.3% was due to violent events, 6.0% to general injury, and 4.1% to falling from a height, while the most preventable cause, traffic accidents, accounted for 15.9%. In all these cases, OHCA can be defined as preventable, as the probability of occurrence can be mitigated with a social control system, proper mobility training, or through increased road security. Thus, the total events occurring in preventable forms was 39.4%. In contrast, an acute medical event, which is unpreventable, was present in 55.2% of cases. This finding, enlightening traumatic events as a leading cause of pediatric OHCA, is in line with statistical analyses performed in other contexts, such as the UK [37], USA [38], and Japan [39], thus confirming that the results are generalizable and extendible. 

According to the available information according to the Utstein model, the data show that in 27.9% of the cases, the OHCA was not witnessed, which makes resuscitative maneuvers less effective and, consequently, the achievement of ROSC more difficult. Many of these cases could be related to Sudden Infant Death Syndrome (SIDS), which mostly affects infants during sleep, thus not giving any bystander a chance to initiate resuscitative maneuvers.

Figure 1 shows the absolute number of OHCAs by age. The curve shows a bi-modal trend of diagnoses in terms of absolute value, which is a new element compared to current knowledge on the topic. Overall, in the pediatric group, the incidence found in the reference population is 4.5 (95% CI 5.6–3.6) per 100 000, which is in line with the data published by Michelson KA et al. [14], despite their study being focused on Emergency Department discharge diagnoses. The bi-modal trend of the phenomenon is confirmed in Figure 2. In fact, in the 0–4 range, the incidence was 5.7 (95% CI 3.8–8.5), while in the 15–18 range, it was 8.8 (95% CI 6.5–12.5). It is possible to hypothesize that this could be related to two different phenomena for the two groups: in the 0–4 age group, a more significant link with organic causes can be supposed, while in the 15–18 age group, the higher incidence could be related to traffic accidents, as the lower percentage of events at home and the high percentage of injuries or accidents seem to corroborate. 

Pediatric OHCA is a topic of great relevance in our territory, where public opinion has shown significant interest on top of the attention from policy-makers, leading to the drafting of a national standard. The previous standard already made the training of dedicated personnel and the installation of an AED mandatory in sports arenas. As expected, the probability for pediatric patients to receive CPR and defibrillation from PAD in these venues is much higher than others, with OR 8.6 (95% CI 2.4–39.9; *p* = 0.001) and OR 12.0 (95% CI 2.7–52.7; *p* = 0.001), respectively. This finding would support the idea of implementing PAD in schools, where, however, events are so rare (six pediatric patients and a total of 22 patients over four years) that, as of today, it is not possible to assess an eventual increased likelihood of CPR and PAD, with the analysis resulting, respectively, in CPR OR: 2.0; 95% CI 0.4–10.5 with *p* = 0.37, and PAD OR: 6.6; 95% CI 0.70–63.07 with *p* = 0.10. Since many OHCAs (39.4%) are of violent origin, it is necessary to ponder priorities and primary interventions to tackle the epidemiology of this phenomenon. Reflecting on models and systems to mitigate OHCAs in the young and violent events is central to all stakeholders. 

Finally, we must report that 14 patients were candidates for ECMO. This critical figure would deserve specific treatment, but still, in the overview of possible policies to be developed, it should be considered, especially given the young age of the patients.

While significant information still emerged from the analysis, this study is subject to some limitations. The most important one is related to data availability: the analysis is relevant to the period preceding the COVID-19 pandemic, from 2016 to 2019, meaning that data are partly outdated. An extension of the dataset is desirable, in particular, considering that events in schools are so rare (six pediatric patients and a total of 22 patients over four years) that, despite such a low incidence is a relevant information itself, as of today it is not possible to assess if there is a statistically significant increased likelihood of CPR and PAD in that venue. However, as discussed in the introduction, the impact of the pandemic strongly affected the EMS network, and in particular OHCA management, and including these data in the analysis would lead to potentially misleading conclusions with relevance to an ordinary condition. Moreover, the legal framework object of this study is dated 2021 and is therefore the result of a legislative path referring to the pre-pandemic period. A re-assessment of the scenario in the post-pandemic era is one of the main future directions for this research line. In addition to the availability of time, another important limitation in the data is the inavailability of clinical outcome data other than those for ROSC. Unfortunately, in the context of the Lombardy region, clinical data are only retained by hospitals and other healthcare facilities; are not linked to the EMS activities (which is the data source for this study); and have no structured processes to retrieve them, as all the data collection, storage, and processing is handled separately by each hospital administration, with their own procedures and standards. Finally, an additional matter regards data quality, as the source information was not collected specifically for scientific research but is instead generated by manual collection from competent personnel during emergency situations. As a result, for instance, with regards to the Utstein model as applied to understand who made the first finding, we must point out that in 26.7% of the cases, the figure was not reported, which hinders the validity of the Utstein analysis.

Ultimately, it is important to remark that, regardless the spatial distribution of AED, a vital part of PAD programs is training lay rescuers [40,41]. This is a particularly critical issue for the territory of the Lombardy region, where the use of AED in OHCA cases is historically limited [42]. Along with an optimal distribution of AED, campaigns for citizen training and raising awareness must be included in PAD programs to ensure its effectiveness, as increasing spatial coverage and/or accessibility of AED does not automatically imply its increased use. 

## 5. Conclusions

Pediatric cardiac arrest is a critical pathology. To this end, several bills have been promoted in Italy to expand mandatory AEDs for schools and sports arenas. Our analysis shows that most pediatric OHCAs occur in two life stages and are likely related to congenital malformations or, during adolescence, to traffic accidents or violent events, which is in line with other studies. The implementation of specific campaigns to address preventable deaths is essential. Even though the knowledge about the role of traumatic events as a major cause of pediatric OHCA is not new, the further confirmation provided this study (on top of enhancing the general validity of this result) is also relevant to enforce the call for a temporal follow-up on these data, in order to generate additional evidence on the topic. In particular, the proposed analysis provides a better understanding of the phenomenon and valuable insights for local policy-makers. A local analysis (despite confirming more general knowledge) is essential to consider territory-specific variability, including population sensitivity, implemented campaigns, and adherence levels. This overview may be important in the coming years in the reference territory of the Lombardy Region when, following the general framework of the latest laws in force, technical documents and implementation strategies will have to be developed. 

## Figures and Tables

**Table 1:** Reasons for OHCA onset in pediatric patients rescued by the emergency–urgency system in the Lombardy region, Italy, between 1 January 2016 and 31 December 2019.

	Number	Percentage
Acute medical event	174	55.2%
Traffic accident	50	15.9%
Violent event	26	8.3%
Accident/injury	19	6.0%
Fall from a height	13	4.1%
Drowning	5	1.6%
Railroad accident	5	1.6%
Mass event	4	1.3%
Intoxication	1	0.3%
Animal assault	1	0.3%
Unknown	17	5.4%
Total	315	100%

**Table 2:** Places of patient rescue by the emergency–urgency system for pediatric OHCA occurred in the Lombardy region, Italy, between 1 January 2016 and 31 December 2019.

	Number	Percentage
Home	185	58.7%
Road	73	23.2%
Sports facility	14	4.4%
Lake/water	9	2.9%
Rail/metro	8	2.5%
Public office	7	2.2%
Health facility	6	1.9%
School	6	1.9%
Skiing facility	2	0.6%
Mountain/impervious site	1	0.3%
Unknown—not reported	4	1.3%
Total	315	100%

**Table 3:** Rescue response data, according to the Utstein model, for OHCA events in pediatric patients treated by the emergency–urgency system in the Lombardy region, Italy, between 1 January 2016 and 31 December 2019.

OHCA Response	Number	Percentage
Witnessed	124	39.4%
Not witnessed	88	27.9%
Witnessed EMS	19	6.0%
Missing data	84	26.7%
Rescue Time	Mean (minute)	Standard Deviation
Time to first emergency vehicle on scene	11.08	5.3
Time of overall mission duration	65.03	22.7
Resuscitation outcome	Number	Percentage
ROSC	64	20.3%
Dead on site	111	35.2%
Sent to the Emergency Department	131	41.6%
Missing data	9	2.9%

## Data Availability

Data are available on request.
